# A Preliminary Study of the Effect of Hyperadrenocorticism on Calcium and Phosphate Concentrations, Parathyroid Hormone and Markers of Bone Turnover in Dogs

**DOI:** 10.3389/fvets.2020.00311

**Published:** 2020-06-08

**Authors:** Carmel T. Mooney, Robert E. Shiel, Mary Sekiya, Mark Dunning, Eilidh Gunn

**Affiliations:** ^1^Small Animal Clinical Studies, School of Veterinary Medicine, University College Dublin, Dublin, Ireland; ^2^Veterinary Pathobiology, School of Veterinary Medicine, University College Dublin, Dublin, Ireland; ^3^School of Veterinary Medicine and Science, University of Nottingham, Sutton Bonington Campus, Sutton Bonington, United Kingdom

**Keywords:** intact and whole parathyroid hormone, osteocalcin, ICTP, urine Ntx, hyperparathyroidism

## Abstract

Reports on the effects of hyperadrenocorticism (HAC) on bone turnover in dogs are largely confined to radiographic studies. The aim of this study was to more accurately assess bone turnover in dogs with HAC by measuring circulating total and ionized calcium and phosphate concentrations, both intact and whole parathyroid hormone (PTH) concentrations and markers of both osteoblastic (osteocalcin) and osteoclastic [carboxyterminal cross-linked telopeptide of type 1 collagen (ICTP) and urine aminoterminal telopeptide of type 1 collagen (NTX) activity]. Dogs with HAC and a control group were prospectively enrolled for comparison. Results from 49 dogs with HAC were compared with 39 dogs from a hospital control population. Plasma intact and whole PTH concentrations were determined using a human immunoradiometric assay. Serum osteocalcin and NTX concentrations were measured using human enzyme linked immunosorbent assays. Serum ICTP concentration was measured using a human radioimmunoassay. Total calcium concentrations in dogs with HAC (2.67 ± 0.25 mmol/L) were not significantly different than in the control group (2.67 ± 0.14 mmol/L). By contrast, phosphate concentrations were significantly (*P* = 0.0143) higher in dogs with HAC (1.46 ± 0.30 mmol/L) compared to the control group (1.28 ± 0.33 mmol/L). The median intact PTH concentration in HAC dogs was 9.25 (range, 1.34–95.45) pmol/L, which was significantly (*P* < 0.0001) higher than in the control group [median, 3.88 (range, 2.01–10.31) pmol/L]. Whole PTH concentrations were also significantly (*P* < 0.0001) higher in the HAC group [median, 4.61 (range, 0.56–125.16) pmol/L] compared to the control group [median, 1.83 (range, 0.88–6.81) pmol/L]. Serum osteocalcin and urine NTX concentrations were not significantly different between the two groups of dogs. The median ICTP concentration in dogs with HAC was 2.98 (range, 1.15–6.62) ng/mL which was significantly (*P* < 0.0001) lower than in the control dogs [median, 7.30 (range, 3.68–21.25) ng/mL]. Both whole and intact PTH concentrations are increased in dogs with HAC compared to a hospital control population. This does not however appear to be associated with a decrease in bone formation (as assessed by osteocalcin) or an increase in bone resorption (as assessed by ICTP and urine NTX).

## Introduction

Hyperadrenocorticism (HAC) is a relatively common endocrine disease that occurs in middle-aged to older dogs (1). It shares many similarities with the same condition in humans. However, HAC in humans differs from that in dogs with respect to a high incidence of clinically significant osteoporosis (2, 3). This occurs in both the naturally occurring disease and as a consequence of glucocorticoid therapy. The latter is now considered to be the most common form of secondary osteoporosis and third in overall frequency to postmenopausal and senile osteoporosis (4, 5). So severe is the osteoporosis associated with HAC that up to 70% of affected patients suffer pathological fractures and these may precede the diagnosis of HAC by several years (2).

The mechanisms by which glucocorticoids induce osteoporosis are complex and varied and involve both direct skeletal and indirect extraskeletal effects (6). Cortisol inhibits osteoblastogenesis, increases apoptosis of osteoblasts and directly prolongs the lifespan of osteoclasts (4, 5, 7). Hypercortisolism stimulates the expression of receptor activator of nuclear factor κB ligand (RANK-L) and colony stimulating factor 1 (CSF-1) while decreasing the expression of osteoprotegerin (OPG), the net effect of which is to promote osteoclastogenesis (2, 7). Glucocorticoids also have an indirect effect by decreasing insulin-like growth factor 1 (IGF-1) synthesis in osteoblasts further decreasing bone formation (8). The inhibitory effects of glucocorticoids on pituitary gonadotropins are also important (9). Glucocorticoids are known to decrease intestinal absorption of calcium and increase urinary excretion of calcium and phosphate (10). However, the induction of secondary hyperparathyroidism and an unambiguous role for parathyroid hormone (PTH) in the development of osteoporosis is controversial (11). Overall, the dominant feature of glucocorticoid-induced changes is a reduction in bone formation, with a lesser and early effect on bone resorption (11).

Serum and urinary bone biomarkers are frequently used in humans to non-invasively evaluate bone turnover and metabolism. Osteocalcin is an important biomarker of bone formation that is synthesized by osteoblasts and promotes bone mineralization (12). Its concentration is reduced in patients with HAC, of a magnitude mirroring the severity of hypercortisolism (13). As bone is largely composed of type I collagen fibers, its breakdown is associated with accumulation of fragments such as the carboxy- and aminoterminal telopeptides of type I collagen (CTX and NTX) and the carboxyterminal cross-linked telopeptide of type I collagen (ICTP). In humans with HAC, circulating ICTP and CTX, and urine NTX concentrations are variably increased, normal or decreased largely depending on the duration and severity of the disease (14–17).

There is no known association between the development of pathological fractures and HAC in dogs but the possibility of osteoporosis exists. Initial studies assessing bone density and composition by plain film radiography suggested a degree of osteopenia in dogs with HAC (18) but this was later disputed as artifactual because of the increased peak kilovoltage (kVp) required for adequate views in obese dogs (19). Two separate studies have identified decreased bone mineral density by quantitative computerized tomography (QCT) in both experimental and naturally occurring HAC (20, 21). Additionally, there is evidence that calcium and phosphate homeostasis is altered in dogs with HAC. Several studies have depicted significantly higher circulating phosphate concentrations, and higher and lower urinary fractional excretion of calcium and phosphate, respectively, in dogs with HAC compared to healthy dogs and those with non-adrenal illnesses (22–24). Increasing calcium and decreasing phosphate concentrations have been reported following trilostane treatment (25). Canine HAC has also been associated with increased circulating intact PTH concentrations although this is not consistent across studies (23, 24). Unfortunately, intact PTH assays also measure large circulating inactive C-terminal fragments of PTH potentially complicating the interpretation of hyperparathyroidism (26). Finally, urine NTX concentrations are reportedly increased in dogs receiving glucocorticoids, with the severity of the increase related to higher doses and shorter duration of treatment, compared to a control population of age-matched healthy dogs (27).

The aim of this study was to further evaluate the possible existence of hyperparathyroidism in HAC by measurement of both circulating intact and whole PTH concentrations in affected and unaffected dogs. A further aim was to assess the effect of HAC on markers of bone formation and resorption by measurement of serum osteocalcin and ICTP, and urinary NTX concentrations in the same groups of dogs.

## Materials and Methods

### Clinical Cases

Dogs with naturally occurring HAC presenting to University College Dublin Veterinary Hospital (UCDVH) were prospectively included providing they were not currently being treated for that disease. A hospital control population was similarly selected comprising dogs also presenting to UCDVH for a variety of reasons providing they had not received prior glucocorticoid therapy and in which naturally occurring HAC was not being considered for specific investigation. To avoid any effect of growth on PTH concentrations, young dogs were not included (<9 months for small dogs and <18 months for large/giant dogs) (28). Control dogs and those with HAC were excluded if they had an illness likely to affect calcium and phosphate homeostasis such as kidney disease or malignancy-associated hypercalcemia. A diagnosis of HAC, and differentiation of pituitary dependent (PD) from a functional adrenal tumor (AT), was achieved based on supportive clinical and clinicopathological abnormalities, abnormal adrenal function tests, endogenous adrenocorticotropic hormone (ACTH) measurement, abdominal ultrasonography or advanced imaging results and response to treatment, if applicable, as described previously (29). Supportive clinical and clinicopathological signs included, amongst others, polyuria and polydipsia, hepatomegaly, and dermatological changes (30). Adrenal function tests considered supportive of hyperadrenocorticism included a post ACTH stimulation cortisol concentration of >600 nmol/L and/or a 3 or 8 h post dexamethasone cortisol concentration >27.6 nmol/L. In all cases, excess serum and urine samples collected during routine diagnostic investigations were stored at −80°C for future analysis. Excess plasma was used only if blood samples were centrifuged immediately after collection and rapidly stored at −80°C. Approval and exemption from full ethical review was obtained from UCD's Animal Research Ethics Committee (AREC-14-67-Mooney). Informed owner consent was obtained in all cases.

### Clinicopathological Analyses

All biochemical analyses (total calcium and phosphate) were performed at the UCD Veterinary Diagnostic Laboratory within 3–4 h of blood collection using a RX Imola (Randox) biochemistry analyzer. The reference intervals were 2.3–3.0 mmol/L for total calcium and 0.8–1.8 mmol/L for phosphate. Ionized calcium concentrations were measured in heparinized plasma immediately after collection using a Rapidpoint 500 (Siemens Medical Solutions Diagnostics) and a reference interval of 1.1–1.4 mmol/L (31).

Plasma intact and whole PTH concentrations were determined using a commercially available human immunoradiometric assay (IRMA) (Duo PTH Kit, Scantibodies Laboratory) previously used and validated in dogs (32, 33). Serum osteocalcin concentration was measured using a commercially available human enzyme linked immunosorbent assay (ELISA) (N-MID® Osteocalcin, Immunodiagnostic Systems). This assay has been previously reported for use in dogs (34–36). Serum ICTP concentration was measured using a commercially available human radioimmunoassay (RIA) previously validated for use in dogs (Uniq ICTP RIA, Orion Diagnostica) (37). Urine NTX concentrations were measured using a commercially available human ELISA kit (Osteomark NTX Urine ELISA, Abbott) previously validated for use in dogs (38, 39). Assay values were corrected for urine dilution by expression as a ratio to urine creatinine concentration. Final results are expressed as a ratio of urine NTX [expressed in nM bone collagen equivalents per liter (BCE)] to creatinine (in mM). All assays were carried out according to the manufacturers' recommendations. Both intact and whole PTH, osteocalcin, and ICTP assays were carried out in house. Urine samples were shipped frozen on dry ice to Affinity Biomarker Laboratories (Imperial College London) where urine NTX and creatinine measurements took place.

### Data Analyses

Continuous data were tested for normality using the Shapiro-Wilk method. Data that were not normally distributed are reported as median (range), while normally distributed data are reported as mean [±standard deviation (sd)] and non-parametric (Mann Whitney test) and parametric (Student's *t*-test) analyses used for comparison, respectively. Categorical data were analyzed using chi-squared or Fisher's exact tests as applicable. Correlation was assessed using Spearman's correlation coefficient for non-parametric data where the strength of correlation was interpreted as follows: *r* < 0.15, very weak; *r* > 0.15–0.25, weak; *r* > 0.25–0.40, moderate, *r* > 0.4–0.75, strong and *r* > 0.75, very strong. Data from the healthy control dogs and those with various illnesses were analyzed for any differences before being grouped together (results not included).

Samples for which the measured urine NTX concentration was above the upper limit of the assay (3,000 nM BCE) were assigned that value for statistical purposes. A *P* < 0.05 was considered statistically significant. All statistical analyses were carried out using GraphPad Prism (GraphPad Software Incorporated).

## Results

### Clinical Cases

In total, 49 dogs with HAC were enrolled in the study. With the exception of one case, no dog had received medical treatment (either trilostane or mitotane) for HAC. One dog had previously been treated with trilostane but had had all medication withdrawn at least 5 months prior to presentation. The majority (*n* = 40) of the dogs had PD HAC and three had functional ATs. In the remaining six dogs, further diagnostic investigations were not available to discriminate the underlying etiology. The age of one dog was unknown and the remainder had a mean age of 9.3 ± 2.7 years. There were 24 females (20 neutered, three entire and one of unknown status) and 25 males (14 neutered, 10 entire and one of unknown status). There were 35 (71.4%) pedigree dogs comprising 15 different breeds. The remaining 14 (28.6%) were crossbreeds. There were 40 small (<10 kg) to medium (10–25 kg) and 9 large (>25 kg) sized dogs.

Thirty-nine hospital control dogs were enrolled. These dogs presented for a variety of reasons, most of which were mild, none of which were life-threatening and many of which were for elective surgery. The disorders included orthopedic disease (*n* = 12, including cranial cruciate ligament disease, intervertebral disc disease, osteoarthritis, cervical spondylopathy, bilateral medial coronoid disease, prior fibrocartilaginous embolism, intermittent lameness, hip dysplasia), gastrointestinal disease (*n* = 6), previous non-specific illness now resolved (*n* = 3), heart disease (*n* = 3), idiopathic epilepsy (*n* = 2), skin disease (*n* = 2) and one case each of pharyngeal stick injury, ectopic thyroid carcinoma, central diabetes insipidus and urinary tract infection. Seven further cases were healthy blood donors. These dogs had a mean age of 5.4 ± 2.8 years. There were 20 females (18 neutered, two entire), 18 males (12 neutered, six entire) and one where gender was not recorded. There were 32 (82.1%) pedigree animals comprising 24 different breeds. The remaining 7 (17.9%) were crossbreeds. There were 21 small to medium and 18 large to giant sized dogs.

The dogs with HAC were significantly (*P* < 0.0001) older than the hospital control group. There was no significant difference in the distribution of males and females or pedigree and crossbreed between dogs with HAC and controls (*P* = 0.7534 and *P* = 0.3168, respectively). There was a significant (*P* = 0.005) difference in the number of small to medium and large to giant sized dogs with HAC compared to the control group.

### Clinicopathological Data

**Figure 1:**
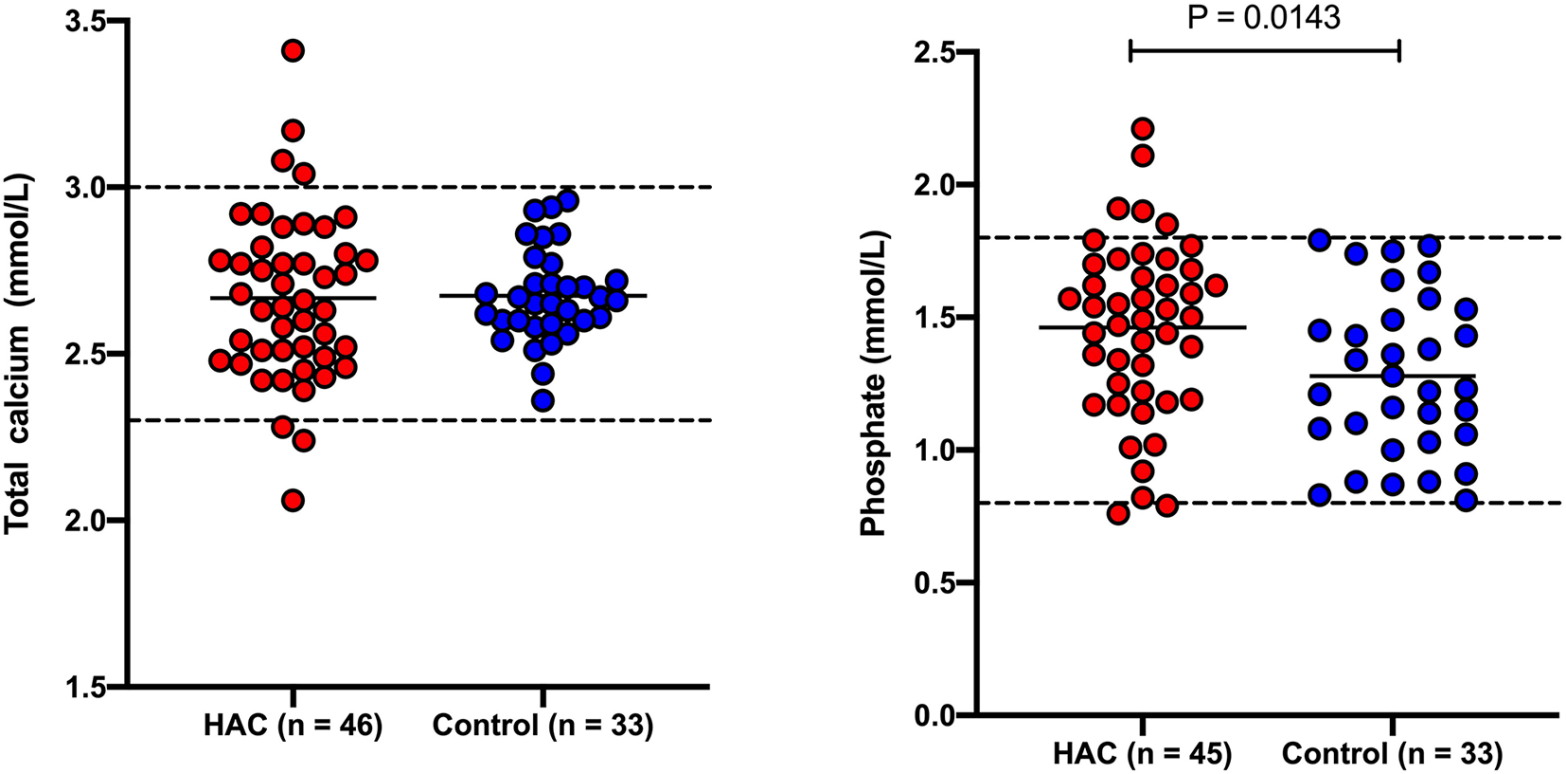
Total calcium and phosphate concentrations in dogs with hyperadrenocorticism (HAC) and a hospital control population. The solid lines intersecting the data represent the mean for each group while the hatched lines represent the respective upper and lower reference limits.

**Figure 2:**
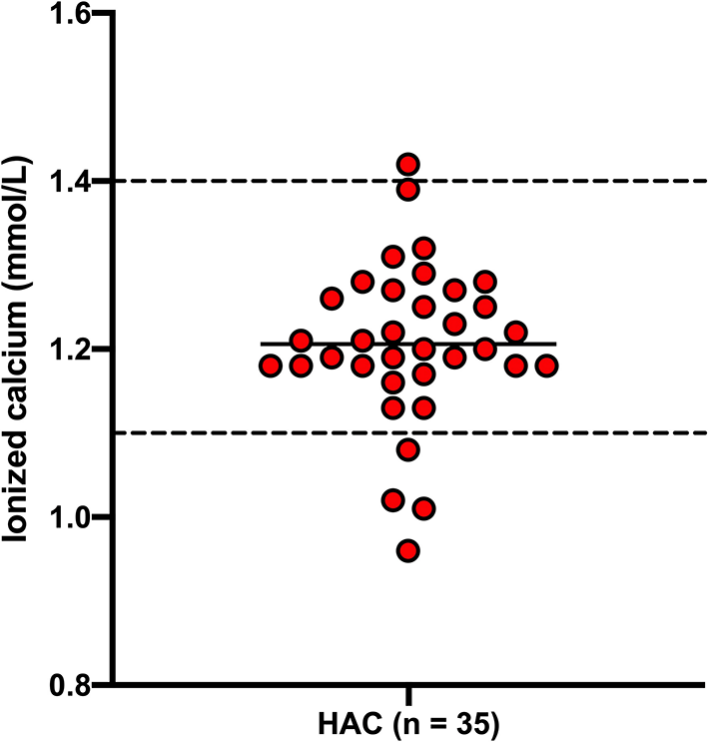
Ionized calcium concentrations in dogs with hyperadrenocorticism (HAC). The solid line intersecting the data represents the mean while the hatched lines represent the respective upper and lower reference limits.

Results for total calcium, phosphate and ionized calcium are presented in Figures 1, 2. Total calcium was high in four (8.7%) of 46 dogs with HAC but the increases were mild to moderate (3.04, 3.08, 3.17, 3.41 mmol/L). Values were mildly decreased (2.06, 2.24, 2.28 mmol/L) in three (6.5%) cases. Ionized calcium was measured in 35 dogs and was 1.21 ± 0.09 mmol/L and included one (2.9%) value (1.42 mmol/L) just above and four (11.4%) values (ranging from 0.96 to 1.08 mmol/L) below the reference interval. Of the two cases with total hypocalcemia, one had an ionized calcium measured that was also low. Three of the four dogs with total hypercalcemia had corresponding ionized calcium measured and all were within reference interval. All 33 of the hospital control dogs, in which total calcium was measured, had reference interval values. Overall total calcium concentrations in dogs with HAC (2.67 ± 0.25 mmol/L) were not significantly (*P* = 0.884) different than values in the hospital control dogs (2.67 ± 0.14 mmol/L). Ionized calcium concentrations were not available from the hospital control dogs. In 45 dogs with HAC where it was measured, phosphate concentrations were above (1.85, 1.90, 1.91, 2.11, and 2.21 mmol/L) and below (0.79 mmol/L) the reference interval in five (11.1%) and one (2.2%) cases, respectively. All 33 of the hospital control dogs, in which phosphate was measured, had reference interval values Overall phosphate concentrations in dogs with HAC (1.46 ± 0.30 mmol/L) were significantly (*P* = 0.0143) higher than in the hospital control dogs (1.28 ± 0.33 mmol/L).

**Figure 3:**
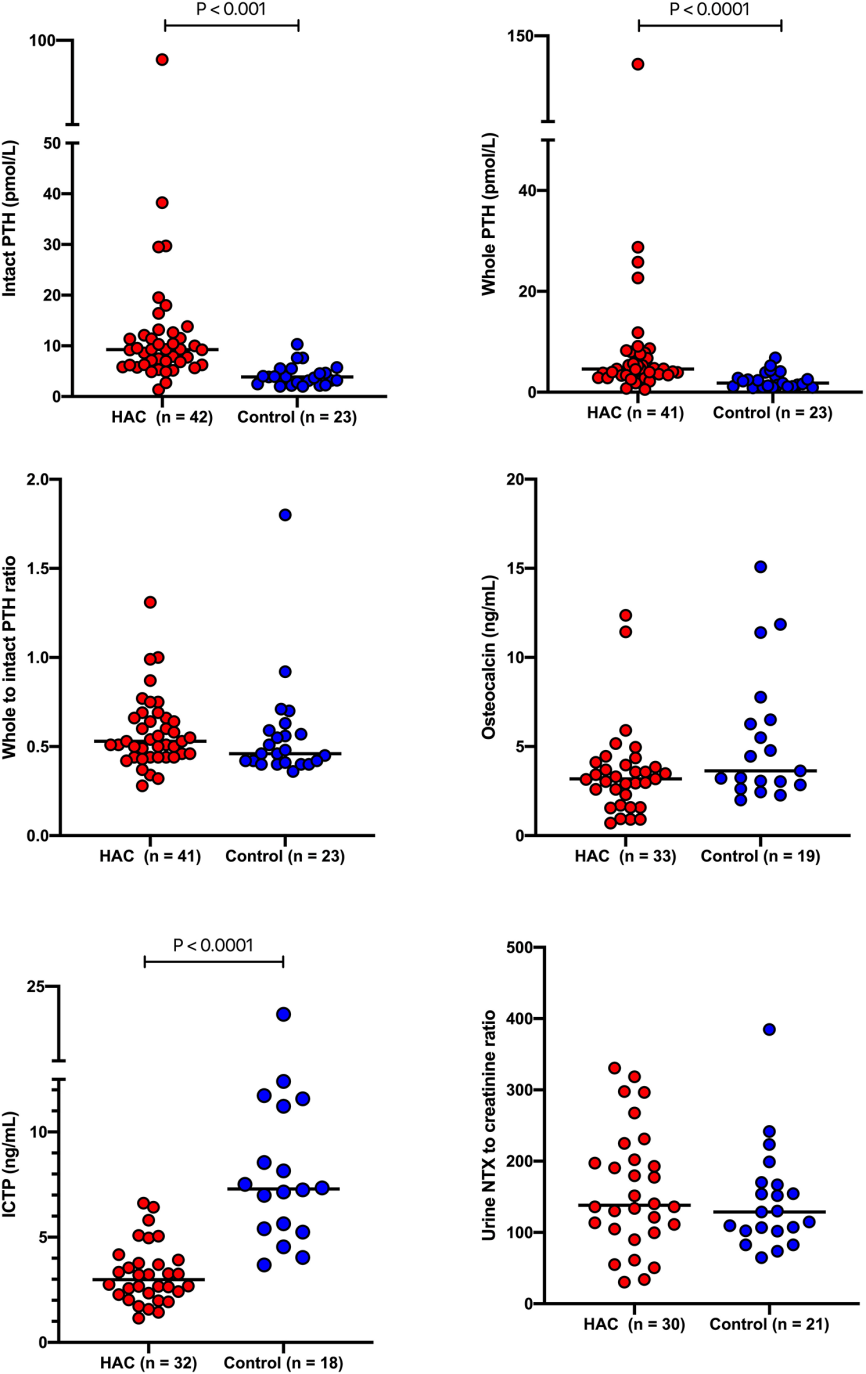
Intact and whole PTH concentrations, the ratio of whole to intact PTH concentrations, osteocalcin and ICTP concentrations and the urine NTX to creatinine ratio in dogs with hyperadrenocorticism and a hospital control population. The solid lines bisecting the data represented the median for each group. PTH, parathyroid hormone; ICTP, carboxy-terminal cross-linked telopeptide of type 1 collagen; NTX, N-terminal cross-linked telopeptide of type 1 collagen; HAC, hyperadrenocorticism.

Results for intact PTH, whole PTH, the ratio of whole to intact PTH, osteocalcin, ICTP and the urine NTX to creatinine ratio are presented in Table 1 and Figure 3. Intact and whole PTH concentrations were significantly (*P* < 0.0001 in each case) higher in dogs with HAC compared to the hospital control dogs. There was no significant difference (*P* = 0.188) in the whole to intact PTH ratio between the two groups. Intact and whole PTH concentrations were very strongly correlated in the HAC and the hospital control groups (*r* = 0.894 (95% CI, 0.8063–0.9437) *P* < 0.0001 and *r* = 0.8518 (95% CI, 0.6704–0.9371) *P* < 0.0001, respectively). In three of the dogs with HAC and one hospital control dog, the whole PTH concentration approached or exceeded the intact PTH concentration (ratios of 0.99, 1.00, 1.31 and 1.8, respectively). There was no significant correlation between intact or whole PTH and phosphate concentrations (*r* = −0.1193, *P* −0.4756 and *r* = −0.155 and *P* = 0.3598, respectively). Serum osteocalcin and urine NTX concentrations were not significantly different (*P* = 0.076 and 0.453, respectively) while serum ICTP concentrations were significantly (*P* < 0.0001) lower in dogs with HAC compared to the hospital control animals.

**Table 1:** Intact and whole PTH concentrations (measured in pmol/L), the ratio of whole to intact PTH concentrations, osteocalcin (ng/mL), and ICTP (ng/mL) concentrations and the urine NTX to creatinine ratio in dogs with hyperadrenocorticism and a hospital control population.

		**Intact PTH**	**Whole PTH**	**PTH ratio**	**Osteocalcin**	**ICTP**	**Urine NTX ratio**
**HAC**	Median	9.25	4.61	0.53	3.18	2.98	160.2 ± 84.1*
	Minimum	1.34	0.56	0.28	0.70	1.15	30.3
	Maximum	95.45	125.16	1.31	12.36	6.62	300.3
**Control**	Median	3.88	1.83	0.46	3.63	7.30	128.8
	Minimum	2.01	0.88	0.36	2.00	3.68	64.8
	Maximum	10.31	6.81	1.80	15.08	21.25	384.6

The number of dogs for each measurement are illustrated in Figure 3. PTH, parathyroid hormone; ICTP, carboxy-terminal cross-linked telopeptide of type 1 collagen; NTX, amino-terminal cross-linked telopeptide of type 1 collagen; HAC, hyperadrenocorticism. *Urine NTX ratio is reported as mean ± sd as it was normally distributed.

## Discussion

The current study demonstrates that circulating intact PTH concentrations are increased in dogs with HAC compared to a hospital control population. Increased values have previously been described in dogs with HAC compared to a similar hospital population (24). Direct comparison between the two studies is difficult, however, because two different assays were used (IRMA and ELISA) and actual values were not provided in the latter study. However, the median intact PTH concentration in dogs with HAC of ~11.0 pmol/L (as extrapolated from the figures) (24) is comparable to the value of 9.25 pmol/L found in the current study. In another study, there was no significant difference in PTH concentrations between healthy dogs and those HAC (23). However, only a small number (*n* = 12) of dogs in each group were included and the median intact PTH concentration, using a similar IRMA, in the healthy dogs of 8.36 pmol/L was higher than the value of 3.88 pmol/L in the hospital control dogs in the current study. Unfortunately, in the present study, the hospital control population was not specifically matched to the dogs with HAC and while there was no significant difference in breed categorization (pedigree vs. crossbreed) or gender status between the two groups, the dogs with HAC were significantly older and smaller in size. By contrast, the healthy dogs were age, and breed matched in the study by Corbee et al. (23) and only included animals with PDHAC, potentially explaining the difference in results. However, Ramsey et al. (24) who also used an age and weight matched control group found no significant difference in PTH concentrations between dogs with PD HAC and functional ATs. Additionally, it is known that ACTH causes an increase in cortisol but not intact PTH in dogs (40) and that treatment of HAC is associated with a decline in intact PTH concentrations, regardless of underlying cause (25). As such it was considered reasonable to include all dogs with HAC together in one group in this study regardless of whether PDHAC or ATs had been diagnosed. It was not possible to match the control dogs and those with HAC in terms of age or breed and this is acknowledged as a weakness of the current study. However, replication of the previously reported significant difference in intact PTH concentrations in the current study suggests that the less than perfect control group was sufficient to demonstrate a difference compared to those dogs with HAC. Whilst further study on the effects of age and breed or size on PTH concentrations is warranted, overall the evidence presented supports that increased intact PTH concentrations occur in dogs with HAC.

To the authors' knowledge this is the first study to describe whole PTH concentrations in dogs with HAC. The whole PTH assay is likely measuring the 1–84 PTH peptide while the intact PTH assay potentially measures both active PTH and shorter C-terminal fragments most of which are probably 7–84 PTH (26, 41). In both dogs with HAC and the hospital control group, intact PTH values were higher than whole PTH values by ~50% as has been described previously in healthy dogs (32). Dogs with HAC had significantly higher whole PTH concentrations than the hospital control population. However, both whole and intact PTH concentrations remained highly and significantly correlated in HAC and the ratio of whole to PTH values was not significantly different to the hospital control population. This implies that HAC has a similar effect on both intact and whole PTH concentrations and that accumulation of large PTH fragments alone is not an explanation for the increased intact PTH concentrations noted in dogs with HAC. In three dogs with HAC, the whole PTH concentration was approximately equal to or exceeded the intact PTH concentration (ratios of 0.99, 1.0, and 1.31). This has been reported previously in humans and when it occurs in secondary hyperparathyroidism, is thought to reflect the severity of the hyperparathyroidism (42). Certainly, in this study, the highest ratio corresponded to the highest intact and whole PTH concentrations measured in dogs with HAC (95.45 and 125.16 pmol/L, respectively). One dog from the hospital control population also had a higher whole than intact PTH concentration (ratio of 1.8). Higher whole PTH concentrations have also been described in humans with parathyroid carcinoma and primary hyperparathyroidism. However, all four dogs with reversed ratios had reference interval plasma total calcium, phosphate and ionized calcium concentrations making such an explanation unlikely in these cases.

Several studies have investigated calcium and phosphate homeostasis as a possible inciting cause or consequence of the hyperparathyroidism reported with HAC. Hyperadrenocorticism is associated with significantly increased phosphate concentrations, reference interval total and ionized calcium concentrations and lower phosphaturia and higher calciuria compared to healthy dogs and hospital control populations (22, 24, 25). In the present study, HAC was associated with significantly higher phosphate concentrations but had no apparent effect on total calcium concentrations, corroborating these previous findings. Ionized calcium concentrations were only measured in dogs with HAC and although occasional low or high values were found these were marginal and unlikely to be of clinical significance as described previously (24). Overall, the evidence suggests that HAC is associated, at least in part, with increased renal retention of phosphate and enhanced urinary excretion of calcium, potentially explaining the predisposition to calcium containing uroliths (43). The hyperparathyroidism observed in HAC is unlikely to cause these changes as its role in calcium homeostasis is to conserve calcium whilst enhancing phosphate excretion; as such increased circulating calcium and decreased phosphate would be expected. On the other hand, the alterations in phosphate and calcium could induce hyperparathyroidism through negative calcium balance, altered vitamin D metabolism, increased phosphate concentration or changes in other phosphatonins. None of these were directly assessed in the current study. However, given that circulating total calcium was not different in dogs with HAC and that ionized calcium concentrations were largely unaffected suggests that even with the increased calcium excretion previously reported (22), a negative calcium balance is not a likely explanation for the increased PTH concentrations observed in dogs with HAC. A direct effect of increased cortisol or a reduced negative feedback effect of vitamin D also seem an unlikely cause of the hyperparathyroidism as hypercalcemia and hypophosphatemia were not observed. Additionally, vitamin D concentrations have previously been shown to be largely unaffected by HAC, although only a small number of dogs were investigated (23). A direct effect of hyperphosphatemia on inducing increased PTH concentrations is possible as postulated in cats (44). However, if this were the case, the fact that there was no correlation between phosphate and PTH concentrations in the current and previous studies (24) is unexpected. Clearly these possibilities warrant further investigation in dogs with HAC.

Whatever its underlying cause, the existence of hyperparathyroidism in HAC has potential implications on bone metabolism in dogs. Although there is no known association between clinically significant osteoporosis and pathological fractures in canine HAC, some QCT studies are suggestive of decreased bone mineral density with naturally-occurring and experimental hypercortisolism (20, 21). However, in the present study there was no significant difference in concentrations of markers of bone formation (osteocalcin) in dogs with HAC compared to the hospital control population. This contrasts to the situation in humans where osteocalcin is significantly decreased with hypercortisolism (13–17). With regard to markers of bone resorption, urine NTX concentrations were unaffected in dogs with HAC in this study. Variable concentrations of urine NTX have been previously demonstrated in humans and postulated to relate to the duration of hypercortisolism (17). In accord with this, it has been shown that exogenous glucocorticoids transiently increase bone resorption but that this decreases again with longer term treatment (45). Indeed, bone loss is reported to be most rapid in humans during the first 1–6 months of glucocorticoid therapy (46, 47). Similarly, increased urine NTX concentrations in dogs treated with glucocorticoids decline over time, with no significant difference from healthy control dogs after 31 days of therapy (27). Given the insidious nature of naturally occurring HAC, it is likely that affected dogs have been hypercortisolemic for weeks or months prior to presentation, potentially explaining why urine NTX concentrations are unaffected.

Surprisingly, serum ICTP concentrations were lower in the dogs with HAC in the present study. This has also been described in humans with HAC and suggested to represent a greater effect of hypercortisolism on bone formation rather than bone resportion (17). This is an unlikely explanation in dogs given that osteocalcin was unaffected by HAC in the present study. Age may have played a role. As previously described, the dogs with HAC were significantly older than the hospital control group. Serum osteocalcin, ICTP and urine NTX concentrations are all higher in younger dogs and this may have affected results (39, 48). Size was also different between the groups but there is limited information on how this would affect ICTP concentrations. A failure to demonstrate increased ICTP concentrations does not completely rule out an effect of HAC on bone resorption. Release of ICTP is dependent on matrix metalloproteinase or trypsin digestion of bone (49). By contrast CTX is the specific product of cathepsin K-mediated bone resorption, and may be increased when ICTP is not (50). However, as NTX is also a fragment of type 1 collagen and was not significantly different in the dogs with HAC, it is unlikely that measurement of CTX would have provided any further information.

The results of this study confirm significant hyperparathyroidism in dogs with HAC. However, there was limited evidence of decreased bone formation or increased bone resorption in the population of dogs with HAC tested using selected markers of bone turnover. This contrasts to findings in humans where osteoporosis is a common feature in HAC, usually associated with abnormalities in markers of bone turnover but where hyperparathyroidism is an unlikely mechanism (6, 11). The reasons for the differences between humans and dogs are unclear. It is possible that the hyperparathyroidism induced by hypercortisolism prevents any direct hypercortisolism-induced skeletal effects and subsequent development of osteoporosis. In support of this, PTH has been shown to have a therapeutic effect in osteoporosis in affected human patients (11). Alternatively, dog bone may be inherently resistant to the direct effects of prolonged excess glucocorticoids.

This study has many limitations, not least the small sample size. None of the assays used were fully validated at the authors' laboratory but all have been used in previous studies. Reference limits were not generated for the hormones and markers of bone turnover evaluated. The use of a hospital control population was considered a reasonable alternative and although care was taken to avoid the use of dogs with disorders known to affect calcium and phosphate metabolism and parathyroid function, these could not be entirely ruled out. Additionally, disorders such as osteoarthritis may have been masked in dogs with HAC. As discussed, there was a significant age and size difference between the dogs with and without HAC that could potentially affect results. Additionally, it was not possible to measure all parameters in each dog presented. Lastly, diurnal variation has been reported for serum osteocalcin and ICTP but not urine NTX concentrations (51). Care was not taken in the present study to ensure samples were taken at the same time each day. Whilst this may have influenced the results, both the dogs with and without HAC were treated similarly thus mitigating against any such confounding effects.

In conclusion, this study confirms that both intact and whole PTH concentrations are increased in dogs with HAC. The significant correlation between the two hormones suggests that measurement of either provides similar information. Phosphate concentrations are also increased in HAC but circulating calcium concentrations appear to be largely unaffected. Despite the existence of hyperparathyroidism, there was no evidence of reduced bone formation or increased bone turnover as assessed by measurement of serum osteocalcin and ICTP, and urine NTX in this population of dogs with HAC. Further studies are required in dogs to understand adrenal secondary hyperparathyroidism and to elucidate the reasons for the apparent resistance to the osteoporotic effects of hypercortisolism in dogs.

## Data Availability Statement

The datasets generated for this study are available on request to the corresponding author.

## Ethics Statement

The animal study was reviewed and approved by University College Dublin Animal Research Ethics Committee. Written informed consent was obtained from the owners for the participation of their animals in this study.

## Author Contributions

CM wrote the paper and supervised the study. RS helped with study design and contributed to writing the paper. MS performed the ELISAs and contributed to writing the paper. MD was instrumental in measuring urine NTX and contributed to writing the paper. EG carried out the research and contributed to writing the paper.

## Conflict of Interest

The authors declare that the research was conducted in the absence of any commercial or financial relationships that could be construed as a potential conflict of interest.
